# Dermoscopy in synchronous melanomas: a case series^☆^

**DOI:** 10.1016/j.abd.2021.05.019

**Published:** 2022-07-07

**Authors:** Daniel Coelho de Sá, Juliana Abreu Pinheiro, Emmanuel Pereira Benevides Magalhães, Maria Araci de Andrade Pontes

**Affiliations:** a Centro de Dermatologia Dona Libânia, Fortaleza, CE, Brazil; b Universidade de Fortaleza, Fortaleza, CE, Brazil

Dear editor,

About 5% of the patients diagnosed with melanoma will have a second primary melanoma, and it is estimated that 26% to 40% of them are synchronous.^1^ Synchronous tumors are defined as those diagnosed at the same time or within a three-month interval.^2^

Melanomas can exhibit a broad spectrum of dermoscopic presentations and the pattern in synchronous cases has been evaluated in only a few studies. It has been suggested that if endogenous and exogenous factors for an individual remain the same, the clinical and dermoscopic features of the lesions should be similar.^3^ Most of the data come from two publications by Moscarella et al., who evaluated the dermoscopy of multiple melanomas and included 32 patients with synchronous neoplasms in one study and 18 cases in the other.^2^, ^3^ The first study showed that synchronous lesions were more likely to exhibit similar dermoscopy when compared with metachronous melanomas.^3^ In the other study, dermoscopic similarity was correlated with advanced age and photodamage, not being related to synchronicity.^2^ In addition to these studies, four reports of patients with synchronous melanomas were identified in studies that compared the dermoscopy of the lesions.^4^, ^5^, ^6^, ^7^

A retrospective study of patients with synchronous primary cutaneous melanomas attended between July 2016 and December 2019, is presented here, comparing the lesions regarding the histopathological, clinical, and dermoscopic aspects. This is the first study carried out in a South American population.

Eight patients with synchronous melanomas were identified (five had two melanomas each, two had three melanomas and one had four), totaling 20 melanomas. The age ranged between 53 and 73 years, in five men and three women. Regarding the histopathological type, there were 14 superficial spreading, four lentigo maligna, one nodular, and one melanoma with mixed characteristics. Fifteen were classified as *in situ* melanomas and five as invasive. Nine lesions (45%) were not considered suspicious after anamnesis and physical examination, but all were classified as at-risk lesions after dermoscopy (Table 1). Table 2 describes the dermoscopic findings.

**Table 1:** Clinical and histological aspects of melanomas per patient.

**Patients**	1	2	3	4	5	6	7	8
**Sex**	F	M	M	F	F	M	M	M
**Age**	65	53	63	69	59	71	67	73
**Location**	A. Right shoulder - lateral aspect	A. Right forearm	A. Right arm	A. Right scapular region	A. Left arm	A. Left scapular region	A. Right arm	A. Right shoulder
	B. Right shoulder - medial aspect	B. Left arm	B. Left zygomatic region	B. Dorsum, left side	B. Right thigh	B. Right scapular region	B. Left trapezius	B. Left interscapular region
	C. Upper left scapular region					C. Sternal region		C. Right interscapular region
	D. Lower left scapular region							
**Clinically suspicious lesion**	A: Yes	A: Yes	A: Yes	A: Yes	A: Yes	A: Yes	A: Yes	A: No
	B: No	B: No	B: No	B: Yes	B: Yes	B: No	B: Yes	B: No
	C: Yes					C: No		C: No
	D: No							
**Histopathological type**	A. Lentigo maligna	A. Superficial spreading	A. Nodular	A. Superficial spreading	A. Superficial spreading	A. Superficial spreading	A. Superficial spreading	A. Superficial spreading
	B. Lentigo maligna	B. Superficial spreading	B. Lentigo maligna	B. Superficial spreading	B. Superficial spreading	B. Superficial spreading	B. Superficial spreading	B. Superficial spreading
	C. Lentigo maligna					C. Superficial spreading		C. Superficial spreading
	D. Mixed characteristics of lentigo maligna and superficial spreading							
**Breslow**	A. *In situ*	A. 0.1 mm	A. 1 mm	A. *In situ*	A, 1.3 mm	A. *In situ*	A. 1.2 mm	A. *In situ*
	B. *In situ*	B. *In situ*	B. *In situ*	B. *In situ*	B. 0.3 mm	B. *In situ*	B. *In situ*	B. *In situ*
	C. *In situ*					C. *In situ*		C. *In situ*
	D. *In situ*							
**Ulceration**	A. Absent	A. Absent	A. Absent	A. Absent	A. Present	A. Absent	A. Absent	A. Absent
	B. Absent	B. Absent	B. Absent	B. Absent	B. Absent	B. Absent	B. Absent	B. Absent
	C. Absent					C. Absent		C. Absent
	D. Absent							
**Staging**	A. 0	A. IA	A. IB	A. 0	A. IIA	A. 0	A. IB	A. 0
	B. 0	B. 0	B. 0	B. 0	B. IA	B. 0	B. 0	B. 0

**Table 2:** Description of dermoscopy.

Patient	Dermoscopic characteristics of the lesions, identified by letter for each patient.
Patient 1	A. Asymmetry in the two axes; four colors (light brown, dark brown, gray, black); brown angulated lines; gray veil; few irregular brown and black dots.
	B. Asymmetry in the two axes; two colors (light brown, dark brown); structureless brown area occupying most of the lesion; few non clustered brown dots; discrete brown angulated lines.
	C. Asymmetry in the two axes; five colors (white, light brown, dark brown, red, black); white structureless areas; shiny white structures; structureless, dark brown and black area; atypical pigment network.
	D. Asymmetry in the two axes; two colors (light brown and gray); gray angulated lines; negative network; structureless light brown area; pigment network.
Patient 2	A. Asymmetry in the two axes; five colors (blue, light brown, dark brown, red, black); bluish veil; irregular dots and globules; structureless brown area.
	B. Asymmetry in the two axes; one color (light brown); pseudopods and irregular globules on the periphery; central, structureless light brown area (spitzoid pattern).
Patient 3	A. Asymmetry in the two axes; five colors (blue, white, dark brown, red, yellow); bluish gray veil; shiny white structures; hairpin vessels and irregular linear vessels (atypical vascular pattern); structureless dark brown area.
	B. Asymmetry in the two axes; two colors (light brown and dark brown); asymmetric perifollicular pigmentation. irregular dots and globules.
Patient 4	A. Asymmetry in the two axes; four colors (gray, light brown, dark brown, white); gray veil; angulated lines; white scar areas; atypical pigment network.
	B. Asymmetry in the two axes; five colors (light brown, dark brown, red, white, gray); white structureless areas; angulated lines; atypical pigment network; irregular linear vessels.
Patient 5	A. Asymmetry in the two axes; six colors (blue, black, light brown, dark brown, white and red); whitish blue veil; atypical pigment network; ulceration.
	B. Asymmetry in the two axes; four colors (light brown, dark brown, red, white); white structureless area; irregular dots and globules; atypical pigment network; eccentric, dark brown structureless area from which pseudopods protrude into the lesion (dermoscopic island); shiny white structures; irregular linear vessels.
Patient 6	A. Asymmetry in the two axes; five colors (black, blue, light brown, dark brown, red); bluish veil; atypical pigment network; irregular striations; irregular dots.
	B. Asymmetry in the two axes; four colors (white, red, grey, light brown); white structureless area; gray angulated lines; few irregular brown globules.
	C. Asymmetry in the two axes; four colors (light brown, gray, white, red); gray veil; angulated lines; fine pigment network; peppering; white structureless areas.
Patient 7	A. Asymmetry in the two axes; five colors (red, light brown, dark brown, white, gray); hairpin vessels and irregular linear vessels; irregular dots and globules; structureless light brown areas; shiny white structures; rosettes; white structureless areas.
	B. Asymmetry in the two axes; two colors (light brown and dark brown); angulated lines; structureless brown area.
Patient 8	A. Asymmetry in the two axes; five colors (light brown, dark brown, white, red, gray); white structureless area; structureless irregular brown area; pigment network; irregular globules; peppering.
	B. Asymmetry in the two axes; four colors (light brown, red, white and gray); white structureless area; structureless brown area; peppering; fine pigment network; irregular linear vessels.
	C. Asymmetry in the two axes; four colors (light brown, dark brown, white, gray); structureless light brown and dark brown areas; branched pigment network; white structureless area; peppering.

All lesions showed asymmetry in the two axes on dermoscopy and most of them exhibited three colors or more (75%). The most prevalent characteristics were structureless areas, pigment networks, dots and/or globules, and white structureless areas. Angulated lines, seen in 40% of melanomas, have not been described in other publications on synchronous melanomas, and comprise structures that have been associated with lentigo maligna.^8^ Three lesions with angulated lines in the same patient showed this subtype, but the other five, identified in three patients, were superficial spreading melanomas. In seven patients, all melanomas were located in areas with signs of chronic severe sun exposure, an environmental factor that may influence dermoscopic features.^2^ Only patient 5 had one of the melanomas (lesion B) in a region without signs of photodamage.

The interpretation of dermoscopic similarity presents subjective and objective variables and faces limitations due to the lack of standardization in the literature and the great interobserver variability. The authors considered there was partial similarity between the two lesions of patient 4 and between the three lesions of patient 8. In patients 1, 5 and 6, the authors considered that, despite sharing some criteria, there was no similarity in the same patient. Complete divergence on dermoscopy was observed in patients 2, 3 and 7.

In patient 1, angulated lines were the most remarkable dermoscopic structure of lesion A, whereas they were discreetly present in lesion B, absent in lesion C and present in lesion D (Fig. 1A–D). The similarity was due to staging, histopathological type, and anatomical location.

**Figure 1:**
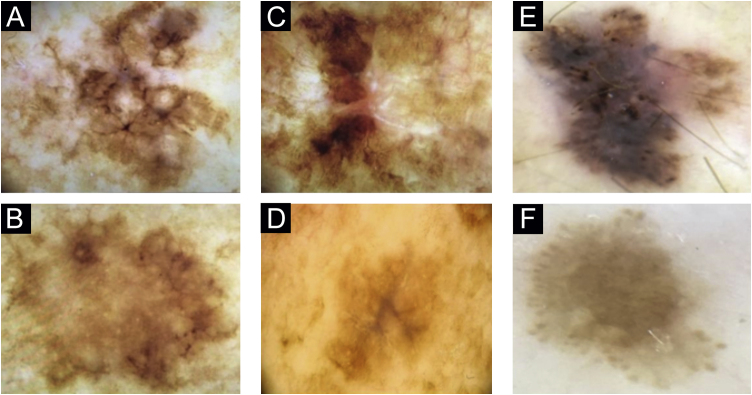
Dermoscopy. (A‒D), The four lesions of patient 1. (A), Right shoulder, lateral aspect. (B), Right shoulder, medial aspect. (C), Upper left scapular region. (D), Lower left scapular region. (E and F), The two lesions of patient 2. (E), Right forearm. (F), Left arm.

Two lesions with different colors and dermoscopic structures were observed in patient 2. Clinically, lesion A was considered suspicious, whereas lesion B seemed benign. The similarity was observed regarding the histopathological type (Fig. 1E–F). Patient 3 showed complete divergence regarding the location, histopathological type, staging, colors, and dermoscopic structures of the lesions. Clinically, lesion A was suspicious because it was a black nodule that had recently appeared, whereas lesion B was a small brownish macula without any dermoscopic risk criteria (Fig. 2A–B).

**Figure 2:**
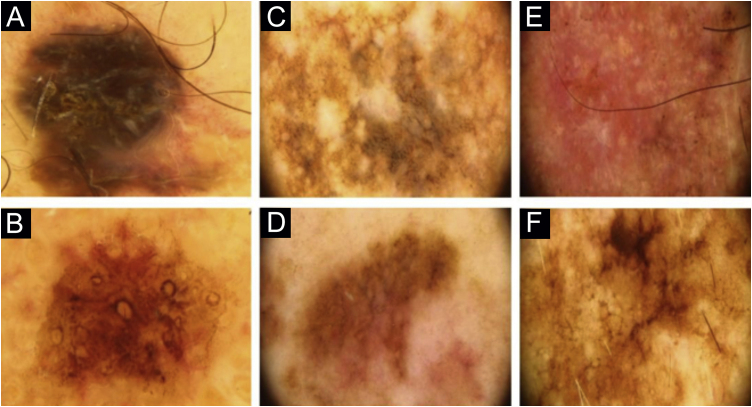
Dermoscopy. (A-B), The two lesions of patient 3. (A), Right arm. (B), Left zygomatic region. (C–D), The two lesions of patient 4. (C), Right scapular region. (D), Dorsum, left side. (E–F), The two lesions of patient 7. (E), Right arm. (F), Left trapezius.

In patient 4, the lesions were located on the back; they had the same clinical appearance, histopathological type and staging. The authors considered there was a reasonably similar dermoscopic appearance, with common structures, such as angulated lines. However, there were differences, such as the presence of gray veil only in lesion A and the vascular pattern only in lesion B (Fig. 2C–D). Patient 5 showed similarity regarding the histopathological type, but divergence concerning the staging. Lesion A of patient 5 was the only one in the study that did not have the dermoscopy image available for review by the authors and so, the medical record description was used, which limited the value of the comparison.

Patient 6 had the same histopathological type and staging for the three lesions. An atypical pigment network and irregular striations were identified only in lesion A. A very evident blue-gray veil was evident in lesion A, more discreet in lesion C and absent in lesion B. Angulated lines were absent in lesion A and present in lesions B and C.

Patient 7 had two clinically different lesions. The histopathological type was the same, but with different staging. The dermoscopic criteria were completely different in the two lesions. Rosettes, seen in lesion A, are rarely described in melanomas (Fig. 2E–F).^9^ Patient 8 had three melanomas with the same histopathological type and all of them were *in situ*. Similarities were observed on dermoscopy of the three lesions, such as regression structures.

The sensitivity of clinical examination for melanoma diagnosis has been estimated at 70% in some studies.^10^ In the present series, only 55% of the lesions were clinically suspicious. The limited sample size prevents the authors from drawing conclusions but raises the hypothesis, to be investigated, that patients with synchronous melanomas might have a greater chance of having lesions that are difficult to be diagnosed.

In the present study, a perfect dermoscopic similarity was not observed between synchronous melanomas in the same patient. Common characteristics were found in some cases. A similar aspect in the same patient could be a complicating factor in lesions considered to be of intermediate risk, as it could lead to misinterpretating the pattern as the patient nevus identity.

Although the present study describes one of the largest series of patients with synchronous melanomas, the sample size is still small, which does not allow the authors to extrapolate the data to other cases.

## Financial support

None declared.

## Authors' contributions

Daniel Coelho de Sá: Statistical analysis; approval of the final version of the manuscript; design and planning of the study; drafting and editing of the manuscript; collection, analysis, and interpretation of data; effective participation in research orientation; intellectual participation in the propaedeutic and/or therapeutic conduct of the studied cases; critical review of the literature; critical review of the manuscript.

Juliana Abreu Pinheiro: Statistical analysis; approval of the final version of the manuscript; design and planning of the study; drafting and editing of the manuscript; collection, analysis, and interpretation of data; critical review of the literature; critical review of the manuscript.

Emmanuel Pereira Benevides Magalhães: approval of the final version of the manuscript; collection, analysis, and interpretation of data; intellectual participation in the propaedeutic and/or therapeutic conduct of the studied cases; critical review of the manuscript.

Maria Araci de Andrade Pontes: Approval of the final version of the manuscript; design and planning of the study; effective participation in research orientation; critical review of the manuscript.

## Conflicts of interest

None declared.
